# Attenuation of Perfluorooctane Sulfonate-Induced Steatohepatitis by Grape Seed Proanthocyanidin Extract in Mice

**DOI:** 10.1155/2020/8818160

**Published:** 2020-12-09

**Authors:** Tao Huang, Yurong Zhang, Wenjuan Zhang, Tingting Lin, Luoting Chen, Bei Yang, Lei Wu, Jianhua Yang, Dalei Zhang

**Affiliations:** Department of Physiology, Medical College of Nanchang University, Nanchang 330006, China

## Abstract

Perfluorooctane sulfonate (PFOS), an environmentally persistent pollutant, has been revealed to elicit hepatic toxicity. In the current study, we investigated the protective role of grape seed proanthocyanidin extract (GSPE) against PFOS-caused steatohepatitis in mice. Animals were exposed intragastrically to PFOS (10 mg/kg/day), GSPE (150 mg/kg/day), or their combination. After 21 days of treatment, mice exposed to PFOS exhibited steatosis, oxidative stress, and inflammation in the liver. Nevertheless, simultaneous administration of GSPE resumed the declined serum hepatic enzyme activities and histological abnormalities in PFOS-exposed mice. Furthermore, GSPE supplementation reduced the contents of triglyceride (TG) and total cholesterol (TC) and expression of lipid metabolism-associated genes *CD36* and fatty acid-binding protein 4 (*FABP4*) in the liver of mice treated with PFOS. Moreover, GSPE suppressed the generation of lipid peroxidative product malondialdehyde and restored the activity of superoxide dismutase in the liver of PFOS-exposed mice. In addition, GSPE repressed the PFOS-induced hepatic overproduction of proinflammatory cytokines interleukin-6 (IL-6) and tumor necrosis factor-*α* (TNF-*α*). Our results demonstrate that GSPE attenuates PFOS-caused steatohepatitis in mice by regulating lipid metabolism, oxidative stress, and inflammatory response.

## 1. Introduction

Steatohepatitis is a common histological finding that involves a variety of etiologies. Toxicant-associated steatohepatitis is one of the most frequent causes of steatohepatitis [1]. Perfluorooctane sulfonate (PFOS), a member of a family of perfluoroalkyl substances (PFAS), has widely aroused public attention due to its ubiquitous distribution, environmental persistence, high bioaccumulation, and potential toxicity [2]. PFOS has been detected in populations worldwide [3], and the consumption of contaminated foods and drinking water and inhalation of indoor dust are the predominant exposure pathways of PFOS to nonoccupationally exposed people [4]. Owing to the long half-life of elimination in people (approximately 5 years) [5], PFOS accumulates in the body and poses a threat to human health, particularly for occupationally exposed persons. Tests in rodents have revealed that PFOS exposure caused hepatic steatosis [6–9]. In humans, monitoring studies have also reported that serum PFOS is significantly associated with dyslipidemia [10, 11]. Furthermore, PFAS exposure is correlated with increased risk of steatohepatitis and fibrosis in children diagnosed with nonalcoholic fatty liver disease (NAFLD), suggesting that PFOS may be a toxicant contributing to nonalcoholic steatohepatitis (NASH) [12].

Nutrition is a powerful protector against environmental chemical insults [13]. Proanthocyanidins are a class of polyphenols that possess a wide range of health benefits [14]. Grape seed proanthocyanidin extract (GSPE) is a major source of proanthocyanidins. The physiological activities of proanthocyanidins include reduction in oxidative stress, inflammation, and metabolic syndrome which provide multiorgan protection from various drug- and chemical-induced toxicities [15, 16]. Although several studies have shown the protective effect of GSPE on hepatotoxicity induced by diverse environmental pollutants [17–21], it remains unknown whether GSPE exerts hepatoprotection against PFOS-caused liver damage. This study hypothesizes that GSPE can attenuate PFOS-evoked steatohepatitis via improving hepatic lipid metabolism, oxidative stress, and inflammation.

## 2. Materials and Methods

### 2.1. Experimental Animals

Specific pathogen-free male Kunming mice weighing 20 to 22 g were procured from the Laboratory Animal Science Department of Jiangxi University of Traditional Chinese Medicine. The mice were housed at 25°C on a light/dark cycle of 12 h : 12 h with unrestricted diet and water intake. All procedures for animal experimentation were conducted in compliance with the guidelines of Nanchang University Laboratory Animal Research Ethics Committee (no. 20130916).

### 2.2. Treatments

After 1-week adaptation to the laboratory environment, the randomly selected mice were intragastrically treated once daily with PFOS (10 mg/kg/day, ≥98%, Sigma-Aldrich, USA), GSPE (150 mg/kg/day, ≥95%, Sciphar Biotechnology, China), or their combination, with 6 mice in each group. PFOS and GSPE were dissolved in distilled water. Control animals received only the equivalent volume of water. The doses of PFOS and GSPE were chosen based on the reported studies [6, 9, 20, 21]. After 21 consecutive days of administration, mice were weighed and fasting blood samples were drawn under anesthesia from the retroorbital venous plexus for liver function analysis. The mice were sacrificed by cervical dislocation, and the livers were excised, weighed, and frozen in liquid nitrogen for biochemical measurement or fixed with 10% buffered formalin for histological evaluation.

### 2.3. Biochemical Analysis

Activities of lactate dehydrogenase (LDH), alanine transaminase (ALT), and aspartic acid transaminase (AST) in serum and contents of triglyceride (TG) and total cholesterol (TC) in homogenized livers were detected utilizing the colorimetric assay kits acquired from Jiancheng Bioengineering Institute (China).

### 2.4. Histopathology

The formalin-fixed liver specimens were dehydrated in alcohol, infiltrated with paraffin, sliced at 5 *μ*m thickness, and stained with hematoxylin-eosin to observe hepatic morphological changes.

### 2.5. Real-Time Quantitative PCR

Liver RNA obtained by TRIzol was converted to cDNA with a cDNA synthesis kit purchased from TaKaRa Biotechnology (China). Quantitative SYBR Green PCR reactions were conducted using an ABI Prism 7500 apparatus. The primers used for amplification were designed as follows: *CD36*: 5′-GCCAAGCTATTGCGACATGA-3′ (forward), 5′-GGCATTGGCTGGAAGAACAA-3′ (reverse); *FABP4*: 5′-CTTTGTGGGAACCTGGAAGC-3′ (forward), 5′-ATGATCATGTTGGGCTTGGC-3′ (reverse); *GAPDH*: 5′-GGCAAATTCAACGGCACAGT-3′ (forward), 5′-GTCTCGCTCCTGGAAGATGG-3′ (reverse). The mRNA expression of *CD36* and *FABP4* in each sample was normalized to the corresponding *GAPDH*. Relative quantification was calculated using the 2^−*ΔΔ*CT^ method.

### 2.6. Oxidative Stress Assessment

Malondialdehyde (MDA) and hydrogen peroxide (H_2_O_2_) generation and superoxide dismutase (SOD) activity in the liver homogenates were determined to assess hepatic lipid peroxidation, oxidant damage, and antioxidative defense using the colorimetric detection kits supplied by Jiancheng Bioengineering Institute (China).

### 2.7. Proinflammatory Cytokine Determination

The hepatic contents of proinflammatory cytokines interleukin-6 (IL-6) and tumor necrosis factor-*α* (TNF-*α*) were determined using commercial ELISA test kits (Boster Biological Technology, China) following the guidance of the manufacturer.

### 2.8. Statistical Analysis

Experimental data were analyzed statistically by the one-way analysis of variance and Tukey's post hoc comparisons using GraphPad Prism 8.2.1 software. The results were given as means ± SD of 4 mice. *P* value <0.05 was defined as statistically significant.

## 3. Results

### 3.1. GSPE Lowered Liver Index and Serum Liver Enzyme Activities in Mice Exposed to PFOS

**Figure 1:**
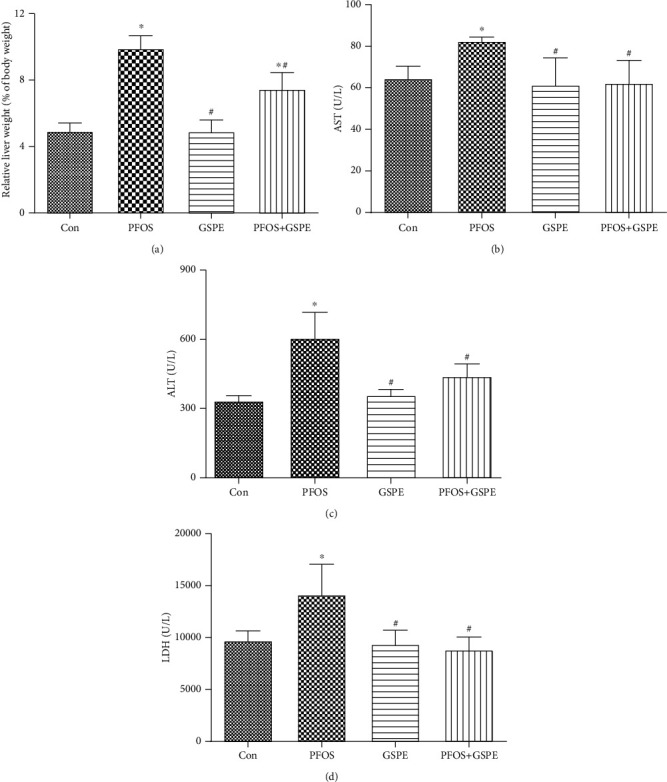
Effects of GSPE on relative liver weight and serum AST, ALT, and LDH activities in PFOS-exposed mice. Values are mean ± SD. ^∗^*P* < 0.05 compared to the control group; ^#^*P* < 0.05 compared to the PFOS group.

To observe the ameliorative role of GSPE in PFOS-caused hepatic injury, liver function parameters AST, ALT, and LDH were detected in mice. As illustrated in Figure 1, intragastric exposure to PFOS for 21 consecutive days resulted in a conspicuous elevation in serum AST, ALT, and LDH activities, with an increase in relative liver weight (*P* < 0.05). Nevertheless, the increased liver index and enzyme activities were resumed by combined treatment with GSPE in mice exposed to PFOS (*P* < 0.05). Treatment with GSPE alone did not influence the liver function of mice (*P* > 0.05).

### 3.2. GSPE Improved the Histopathologic Abnormalities in the Liver of PFOS-Exposed Mice

**Figure 2:**
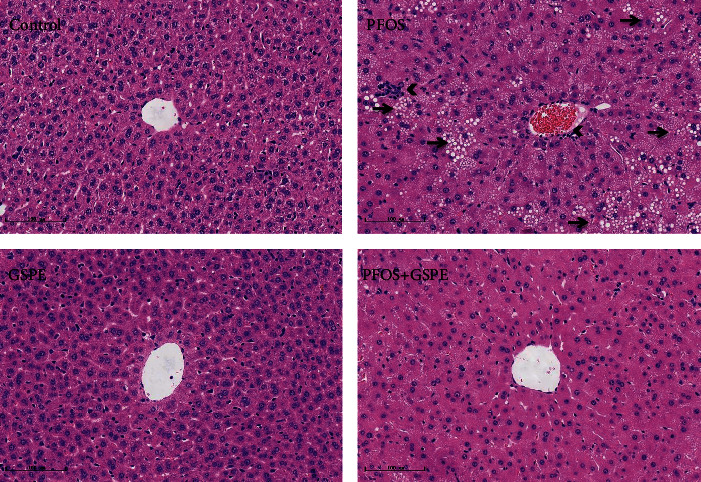
GSPE ameliorated PFOS-induced histological changes in the liver. Arrows indicate steatosis, and arrowheads indicate inflammatory cell infiltration. Scale bar: 100 *μ*m. Magnification: 200x.

As displayed in Figure 2, morphological examination exhibited evident histologic features of steatohepatitis in the liver of mice administrated with PFOS, including architectural disorder, hepatocyte swelling, lipid droplet formation, and inflammatory cell infiltration. However, the pathologic abnormalities were ameliorated by supplementation of GSPE. Mice given only GSPE had the morphological structure of a normal liver.

### 3.3. GSPE Corrected Hepatic Lipid Metabolic Disturbance in Mice Exposed to PFOS

**Figure 3:**
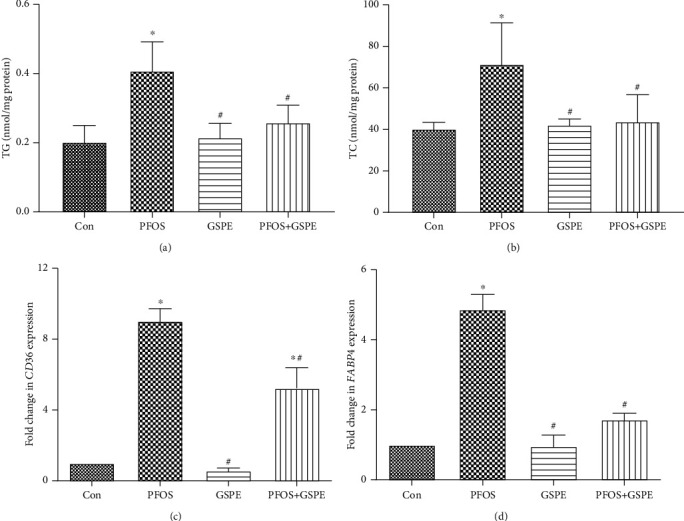
Effects of GSPE on the contents of TG and TC and mRNA expression of *CD36* and *FABP4* in the liver of PFOS-exposed mice. Values are mean ± SD. ^∗^*P* < 0.05 compared to the control group; ^#^*P* < 0.05 compared to the PFOS group.

To further explore the therapeutic effect of GSPE on PFOS-induced steatohepatitis, levels of TC and TG and expression of lipid metabolism-associated genes *CD36* and *FABP4* were analyzed in the liver of mice. As shown in Figure 3, the PFOS challenge induced a marked elevation in hepatic TC and TG contents (*P* < 0.05). Correspondingly, the expression of *CD36* and *FABP4* mRNA in the liver was notably upregulated after 21 days of PFOS exposure (*P* < 0.05). However, the increased lipid content and *CD36* and *FABP4* expression in PFOS-exposed mice were significantly reduced by simultaneous treatment with GSPE (*P* < 0.05).

### 3.4. GSPE Antagonized PFOS-Initiated Oxidative Stress in the Liver

**Figure 4:**
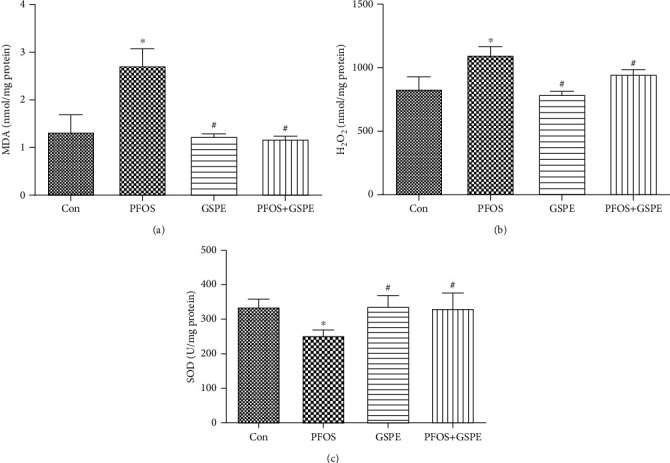
Effects of GSPE on MDA, H_2_O_2_, and SOD levels in the liver of PFOS-treated mice. Values are mean ± SD. ^∗^*P* < 0.05 compared to the control group; ^#^*P* < 0.05 compared to the PFOS group.

As can be seen in Figure 4, compared with the control group, the generation of oxidative stress markers MDA and H_2_O_2_ was enhanced and the hepatic activity of the antioxidant enzyme, SOD, was inhibited in PFOS-administrated mice (*P* < 0.05). Nonetheless, simultaneous GSPE administration reversed the increased levels of MDA and H_2_O_2_ and compensated for reduced SOD activity (*P* < 0.05). GSPE alone did not affect any oxidative stress indicators measured.

### 3.5. GSPE Attenuated PFOS-Caused Inflammation in the Liver

**Figure 5:**
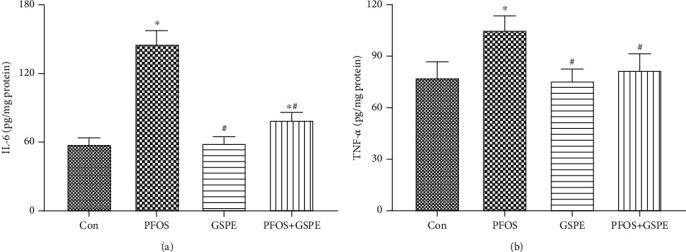
Effects of GSPE on the hepatic expression of IL-6 and TNF-*α* in mice exposed to PFOS. Values are mean ± SD. ^∗^*P* < 0.05 compared to the control group; ^#^*P* < 0.05 compared to the PFOS group.

As presented in Figure 5, significantly increased IL-6 and TNF-*α* expressions were observed in the liver of mice exposed to PFOS compared to the control group (*P* < 0.05). Compared with the PFOS group, the increased IL-6 and TNF-*α* were restored by combined administration with GSPE (*P* < 0.05). GSPE alone did not influence the production of these two inflammatory mediators (*P* > 0.05).

## 4. Discussion

There is increasing evidence that PFOS exerts detrimental effects on health. Considering that the liver is a main target organ for toxic insult arising from PFOS exposure, it is necessary to explore the hepatoprotection against PFOS-induced hepatic damage. Healthful nutrition intervention may be the most advisable strategy against the vulnerability to toxic chemicals. The antioxidant and anti-inflammatory properties of natural bioactive components in functional food can effectively prevent or moderate environmentally associated diseases [13, 22]. In the present study, mice exposed to PFOS exhibited a phenotype of nonalcoholic steatohepatitis, characterized by hepatic steatosis, hepatocyte swelling, and inflammatory infiltrate. Nevertheless, the biochemical and histological abnormalities caused by PFOS were significantly rescued by supplementation with GSPE, implying a hepatoprotective role of GSPE from PFOS-evoked toxicity.

Multiple mechanisms are linked to the pathogenesis and progression of NASH [23]. It has been suggested that PFOS-induced hepatic steatosis involves the disturbance of lipid metabolism [8, 9, 24, 25]. NAFLD is due to excessive fat accumulation in hepatocytes. The lipotoxicity is manifested by hepatocellular ballooning, and triacylglycerols are the major lipid component in ballooned hepatocytes. In NAFLD, increased uptake and accumulation of fatty acids propel the synthesis and storage of triglycerides in hepatocytes [26]. Undue lipid deposition in the liver can exert prooxidative and proinflammatory effects that account for the features of NASH, and the formation of toxic metabolites during lipid metabolism is mainly responsible for the generation of ROS and induction of inflammatory mediators [27, 28].

Grape polyphenols can be proposed as a potential remedy for metabolic syndrome and NAFLD [29, 30]. It has been reported that GSPE ameliorates hepatic lipid metabolism dysfunction in diabetic mice, as well as in rats treated with a high-fat diet, CCl4, and lead [17, 19, 31, 32]. CD36 and FABP4 are key transporters for fatty acid uptake. High hepatic levels of FABP4 have been proposed to be a predictive factor for NASH progression [33]. In NASH mice, FABP4 expression increased in the liver and overexpression of FABP4 in hepatocytes induced an elevation of proinflammatory cytokines. However, the inhibition of FABP4 significantly abrogated NASH-related inflammation [34]. CD36 directly contributes to the development and progression of fatty liver through regulating the uptake of fatty acids by hepatocytes, and the perturbation of hepatic CD36 attenuates fatty liver and associated inflammation in mice fed a high-fat diet [35]. In the present study, PFOS exposure gave rise to lipid dysmetabolism revealed by elevated contents of TG and TC and upregulated expression of lipid metabolic genes *CD36* and *FABP4* in the liver. However, GSPE treatment remarkably reduced lipid accumulation and *CD36* and *FABP4* expression in the liver of PFOS-exposed mice, suggesting that GSPE can reverse hepatic steatosis through modulating lipid metabolism.

Oxidative stress seems to be a critical factor in the pathogenesis of NASH [23]. In the process of hepatic lipid overload, free fatty acids accumulate in the liver and initiate lipid peroxidation. As a consequence of oxidative stress, lipid peroxidation leads to severe injury in the steatotic liver [23]. In NASH patients, lipid peroxidative product MDA in erythrocytes significantly increased, and the oxidative stress in plasma was positively correlated with the severity of steatosis [36]. In this study, PFOS exposure triggered hepatic oxidative stress, which was evidenced by increased MDA generation and declined SOD activity. These results were consistent with previous findings in rodents [37–39]. Likewise, *in vitro* toxicity assessment demonstrated that PFOS increased ROS formation in rat hepatocytes and human HepG2 cells [39, 40]. At present, lifestyle intervention is the main therapeutic option for NASH, and antioxidative treatment may be an attractive candidate [23]. It has been suggested that antioxidant supplementation can ameliorate diet-induced NASH [41]. As a potent free radical scavenger and oxidative inhibitor, GSPE offers stronger protection than vitamin C and E against hepatic lipid peroxidation [42]. Studies have shown that GSPE attenuates fluoride-caused hepatotoxicity by inhibiting oxidative damage in the mouse liver and human embryo hepatocytes [18, 43]. In our experiment, PFOS-induced hepatic oxidative stress and lipid peroxidation were dramatically alleviated by GSPE administration in mice, demonstrating a hepatoprotective potential of GSPE as a natural antioxidant in oxidative damage caused by PFOS exposure.

Inflammation is a hallmark of NASH. It is critical for the development and progression of NASH [27]. Oxidant stress is recognized to exert a pathogenic effect in steatosis-related inflammatory response [44]. Oxidative stress and consequent lipid peroxidation can activate various transcription factors, which lead to an elevation in inflammatory mediators such as TNF-*α*, IL-6, and IL-1. These proinflammatory cytokines are of importance to recruit leukocytes into inflamed tissues [23, 45]. Consistent with previous studies [37, 38], exposure of mice to PFOS in this study resulted in hepatic inflammation manifested by inflammatory cell infiltration, which may be linked to the augmented generation of IL-6 and TNF-*α*. Polyphenols have been proposed as adjuvant therapy for inflammation [45]. It has been shown that GSPE alleviates hepatic inflammation triggered by thioacetamide and lipopolysaccharide via upregulating the expression of proinflammatory factors [46, 47]. In the current experiment, supplementation with GSPE observably repressed the PFOS-induced overproduction of IL-6 and TNF-*α* in hepatic tissue, indicating that GSPE exerted anti-inflammatory activity in mice exposed to PFOS.

## 5. Conclusion

GSPE supplementation can reduce TG and TC accumulation, CD36 and FABP4 expression, MDA and H_2_O_2_ generation, and IL-6 and TNF-*α* production in the liver of mice exposed to PFOS, suggesting that GSPE can serve as a candidate for the prevention and therapy of PFAS-induced steatohepatitis via regulating lipid metabolism, oxidative stress, and inflammatory response. Our results provide a promising pharmacological strategy that may contribute to the protection against toxicant-associated steatohepatitis.

## Data Availability

The data used to support the findings of this study are available from the corresponding author upon request.
